# Does thread shape affect the fixation strength of the bioabsorbable interference screws for anterior cruciate ligament reconstructions? A biomechanical study

**DOI:** 10.1186/s12891-019-2435-1

**Published:** 2019-02-08

**Authors:** Gerardo L. Garcés, Oscar Martel, Alejandro Yánez, Alberto Cuadrado

**Affiliations:** 1 Department of Medical and Surgical Sciences, University of Las Palmas de Gran Canaria, Edificio de Ciencias de la Salud, Campus Universitario de San Cristobal, Trasera del Hospital InsularC/ Doctor Pasteur s/n, 35016 Las Palmas, Spain; 2 Department of Mechanical Engineering, University of Las Palmas de Gran Canaria, Edificio de Ingenierías, Campus de Tafira, 35017 Las Palmas, Spain

**Keywords:** Anterior cruciate ligament, ACL reconstruction, Interference screw, Biomechanical testing

## Abstract

**Background:**

The purpose of this study was to compare the biomechanical behaviour of two bioabsorbable interference screws with different geometries.

**Methods:**

Two different pitch (2.5 and 5 mm) bioabsorbable interference screws, both 9 × 30 mm, were tested. Tests were performed with forty bovine digital extensor tendons and skeletally mature porcine tibiae. Two protocols of cyclic tests at 1 Hz were performed: 1000 cycles from 50 to 250 N, and 5000 cycles from 100 to 300 N (*n* = 10 for each type of test and screw). After the cyclic loading, a final ramp displacement until failure at 0.5 mm/s was applied.

**Results:**

The stiffness after the cyclic phase of the tests was not statistically different between the two screws (1000th cycle: 2.5 mm pitch 280.3 ± 56.4 N/mm, 5 mm pitch 275.2 ± 65.0 N/mm, *P* = .965; 5000th cycle: 2.5 mm pitch 281.3 ± 66.4 N/mm, 5 mm pitch 286.1 ± 79.4 N/mm, *P* = .814). The yield load was not significantly different between the screws (1000 cycle tests: 2.5 mm pitch 482.2 ± 120.2 N, 5 mm pitch 495.9 ± 131.3 N, *P* = .508; 5000 cycle tests: 2.5 mm pitch 476.4 ± 65.3 N, 5 mm pitch 494.3 ± 39.2 N, *P* = .391). No correlation was found between the insertion torque and yield load (1000 cycle tests, *R*^*2*^ = 0.013; 5000 cycle tests, *R*^*2*^ = 0.006).

**Conclusions:**

The pitch of bioabsorbable interference screws does not seem to affect fixation strength. Also, the authors recommend not to use insertion torque alone to estimate the fixation strength.

## Background

The interference screw is the most commonly used fixation device in anterior cruciate ligament (ACL) reconstruction [1]. The screw is manufactured from titanium or a bioabsorbable material, but drawbacks have been reported for both. Bioabsorbable screws have been associated with tunnel widening, risk of screw failure, increased inflammatory response, and incomplete screw absorption, [2] while titanium screws have been associated with graft laceration and hindering magnetic resonance imaging (MRI) capture [3]. Clinical outcomes with titanium and bioabsorbable screws are comparable, [2–5] however the latter offers the additional benefits of allowing MRI, decreasing stress shielding by gradually transferring load during degradation, and theoretically minimizing the difficulty of revision surgery [6]. Therefore, the authors believe that bioabsorbable interference screws appear to be preferable to titanium screws.

Bioabsorbable interference screws are available in different diameters and lengths, with various thread geometries. In a comparative in vitro biomechanical study of different bioabsorbable and titanium interference screws, no differences were found between them [7]. Lately, a high pitch bioabsorbable interference screw, that allows insertion twice as fast as the traditional one, has been introduced. Although some studies have shown that thread geometry does not influence the biomechanical properties of an interference screw, this conclusion was based in one case on magnesium-based screws [8] and in other case on different buttresses screws, but with the same pitch [9].

One possible consequence of a higher pitch is a higher insertion torque, [10] and it is believed that the higher the insertion torque, the better the fixation quality [11, 12]. However, several studies showed that the insertion torque does not predict the strength of the fixation with an interference screw in ACL reconstruction [13, 14]. The effect of screw pitch remains an open issue.

The purpose of this study was to compare the biomechanical behaviour of two bioabsorbable interference screws made of the same material but with different geometries. Our hypothesis was that the two screws had similar in vitro biomechanical properties. A secondary purpose of the study was to determine if a correlation exists between the insertion torque and fixation strength in an ACL reconstruction when bioabsorbable interference screws were used.

## Methods

In this study, forty bovine digital extensor tendons and skeletally mature porcine tibiae were used. Tendons were harvested immediately after the slaughter of the bovines, and they were wrapped in gauze soaked in normal saline and placed in plastic bags. Porcine tibiae used for testing were taken from animals around 6 months old, obtained from a local slaughterhouse after having been sacrificed for human consume. All of them were fed under the same conditions and was assumed that bone density was similar in all the specimens. The fibula and all soft tissue and muscles were removed. The distal end of the tibia was sectioned to attach the bone to a custom-made jaw. Both tendons and bones were stored at − 20 °C. Twelve hours before testing, the tissue was thawed at room temperature and kept moist throughout the handling and testing period.

A custom-made tendon caliper was used to measure the diameter of the grafts. In order to compare both fixation systems in the same conditions, only folded tendons passing the 9-mm bore, but not the 8.5 mm one, were used. Tendon ends were sutured to facilitate handling. During the ACL reconstruction, the bone was attached to a vice. In the tibia, a Ø9-mm bonny tunnel (C-Reamer, Conmed Linvatec, Largo, FL, USA) was created at a 45° angle from the longitudinal axis. The entrance point was the medial side of the tibial tuberosity and the exit point was the upper part of the tibia, approximately at the natural insertion point of the ACL.

**Fig. 1:**
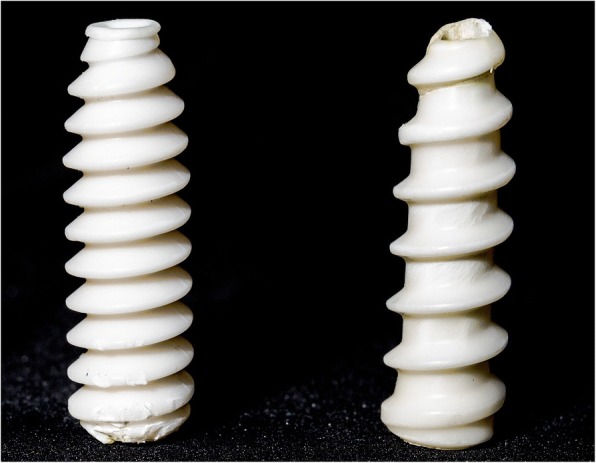
Bioabsorbable interference screws tested. 2.5 mm pitch Milagro (left) and 5 mm pitch Milagro Advance (right)

Two types of 9 × 30 mm bioabsorbable screws from the same manufacturer (DePuy Mitek, Inc. Raynham, MA, USA) were chosen for this study. The 2.5 mm pitch Milagro interference screw and the 5 mm pitch Milagro Advance interference screw were used (Fig. 1). Both screws are made of Biocryl Rapide (30% β-tricalcium phosphate, 70% poly-lactide co-glycolide).

Each reconstruction was performed with a folded tendon with its end sutured to make a double-looped graft. The graft was then inserted into the tunnel with the assistance of the sutures. An approximately 30-mm loop extended out from the upper part of the tibia, simulating the natural ACL intra-articular length [15]. The interference screw was then inserted using a 3.5 mm hex key. During insertion, the loop was fixed, simulating the fe moral fixation and manual tension was applied to the free end of the tendon as in usual surgical routine. The maximum insertion torque was recorded using a digital torque meter (DR-2453, Lorenz Messtechnik GmbH, Alfdorf, Germany) mounted on the hex key.

**Fig. 2:**
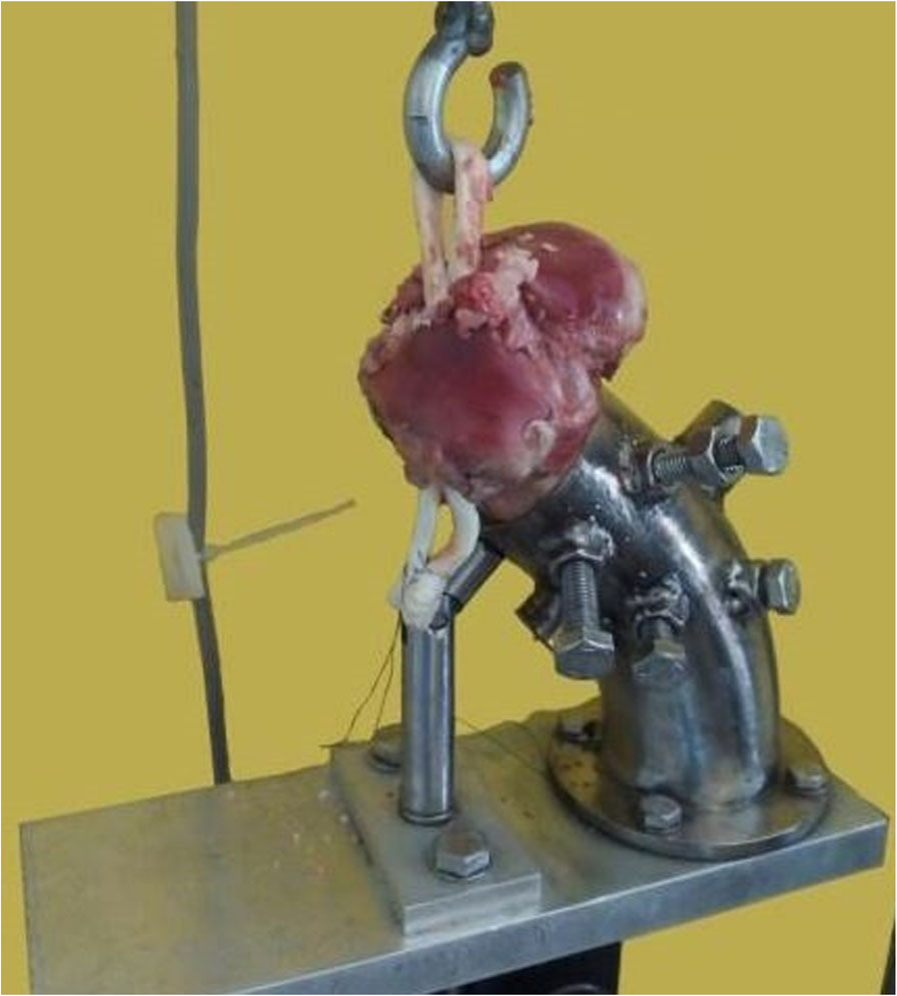
Tibia with the ACL reconstruction performed and inserted in a custom-made jaw mounted at the lower part of the testing machine. The graft loop was attached to the upper grip via a hook

Each specimen was placed on a testing machine (EFH/5/FR, Microtest S.A., Madrid, Spain). The tibia was fixed at the lower part of the machine with a custom-made jaw that holds it at an angle of 45° to the vertical axis and allows it to be pulled in the bonny tunnel direction, which is the worst-case loading scenario for the reconstructed ACL. A hook in the upper grip of the testing machine was used to hold the graft loop (Fig. 2).

Following ACL reconstruction, the specimens were tested. Two types of cyclic fatigue tests with sinusoidal variation in load at 1 Hz were performed: 1) 1000 cycles, 50–250 N, and 2) 5000 cycles, 100–300 N (10 specimens for each type of test and screw). For both type of tests, the minimum load (50 N or 100 N, for tests of type 1 and 2, respectively) was applied for 60 s (s), after which the cyclic testing was performed. The first ten cycles were considered preconditioning. After the cyclic loading, the load was again held at 50 N or 100 N for 60 s and then a final ramp displacement until failure at 0.5 mm/s was applied. In all cyclic tests, a 1 Hz load frequency was used to reproduce normal walking frequency [16].

The 50–250 N load range simulated forces in the ACL during terminal passive extension of the knee [17]. The 1000 cycles approximated one week of flexion-extension loading on an ACL reconstruction [18]. This testing simulated an aggressive, but typical, rehabilitation protocol after an ACL reconstruction [19]. The 300 N load is the upper force expected during normal daily activities, so cyclic testing up to 300 N simulated peaks occurring postoperatively [16]. The 5000 cycles represented an extreme test of the free graft fixation stability [20]. This test represented the worst-case scenario for an ACL reconstruction, i.e., lack of a rehabilitation protocol and early normal daily activities.

**Fig. 3:**
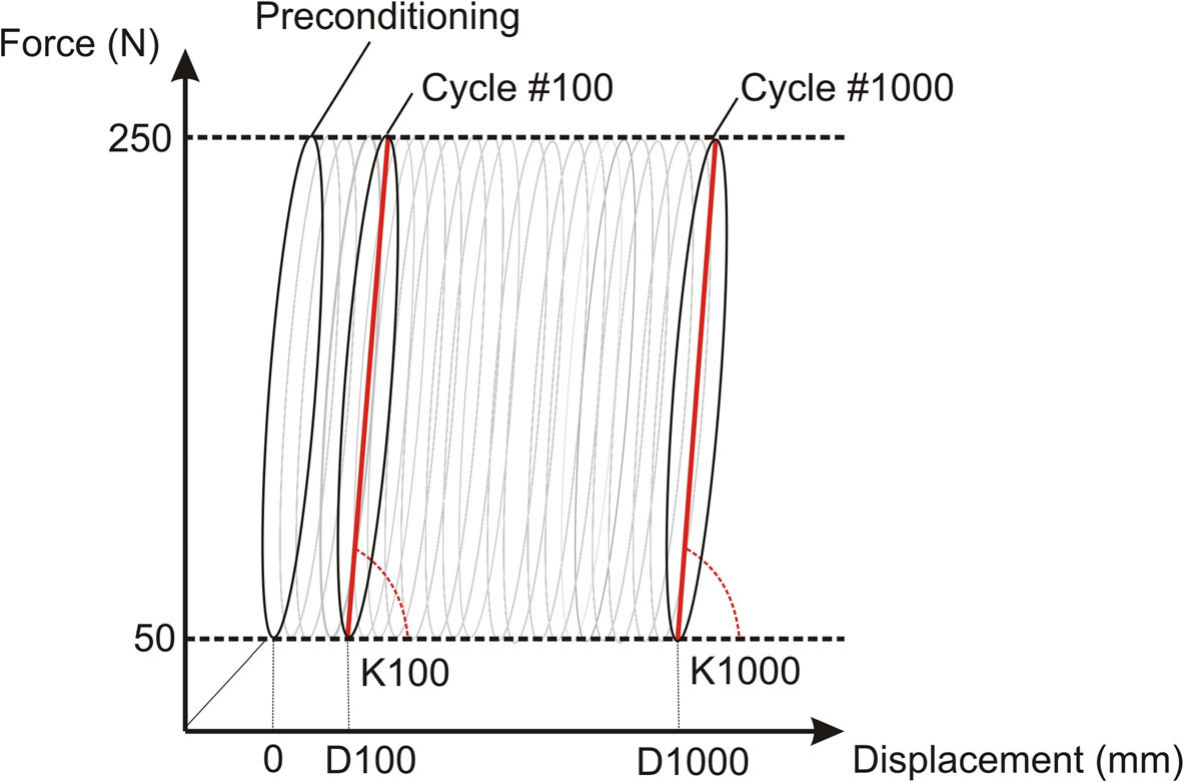
Force vs. displacement plot during cyclic test phase, showing the measured displacement. The stiffness (K) is showed as the slope of the line connecting the maximum and minimum points in a complete cycle

Data from 1 cycle every 100 cycles (for the 1000 cycle tests) or every 500 cycles (for the 5000 cycle tests) were recorded at 100 Hz. Load and displacement were obtained from the load cell and displacement sensor of the testing machine, respectively. In the cyclic phase of each test, stiffness and displacement results were obtained (Fig. 3). Stiffness was defined as the slope of the line connecting the maximum and minimum points of the load-displacement graft in a complete cycle. Stiffness was determined at the 100th and 1000th cycle for the 1000 cycle tests and at the 500th and 5000th cycle for the 5000 cycle tests. Displacement was set to zero after the preconditioning period and was obtained from the same cycles as the stiffness values. All displacements were measured at minimum cyclic load.

**Fig. 4:**
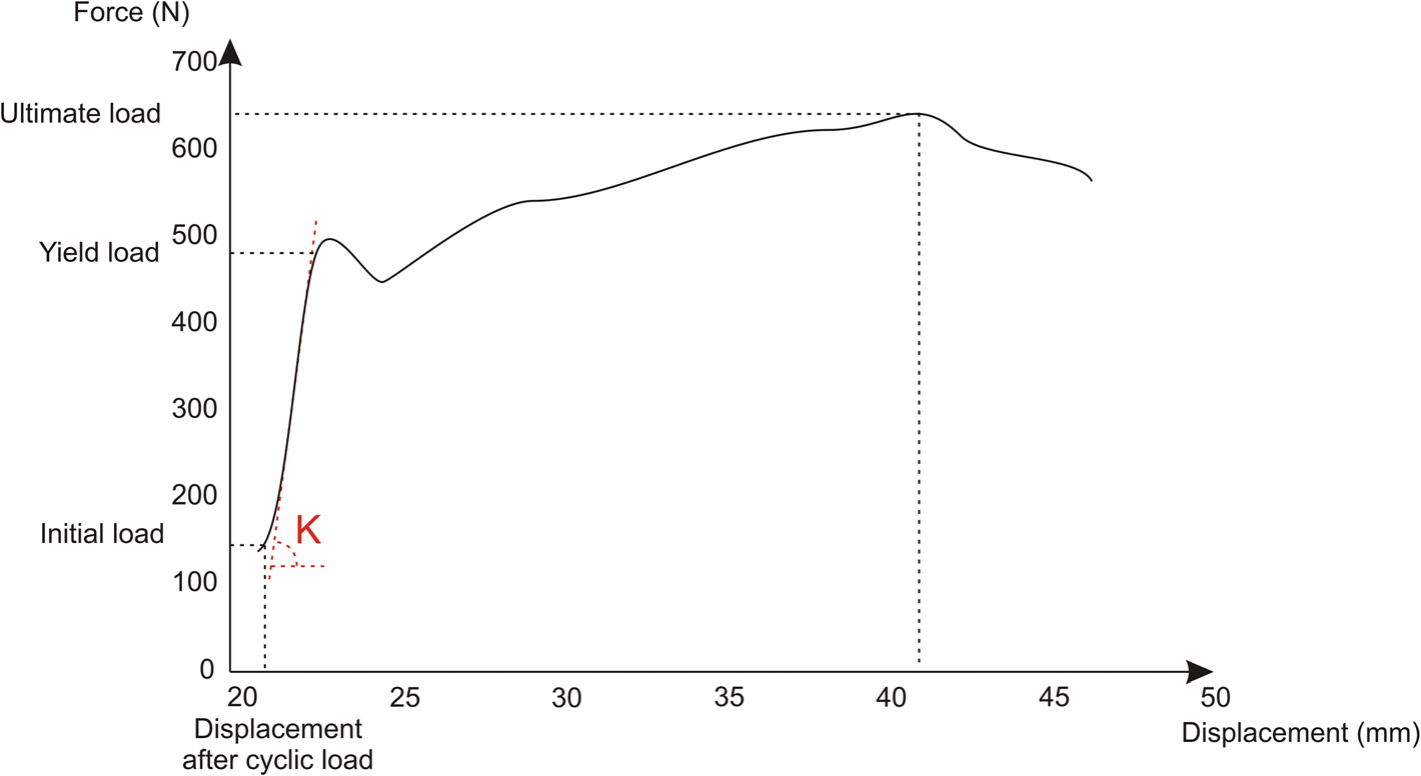
Force vs. displacement plot during final monotonic tensile load, showing yield load, ultimate failure load and pull-out stiffness (K) as the slope of the linear region of the graph

During application of the final monotonic tensile load, pull-out stiffness, yield load, and ultimate failure load were measured (Fig. 4). Pull-out stiffness was determined as the slope of the linear region of the force-displacement curve. Linearity was assumed when Pearson’s correlation was equal to or greater than 0.99. Yield load was determined as the load corresponding to the intersection point of the force-displacement curve and the stiffness determination line offset 0.06 mm, which corresponded to a deformation of 0.2% of the graft length. Ultimate failure load was the maximum load registered during the test.

Data were analyzed using SPSS v 24.0 (IBM, Chicago, IL, USA). An a priori power analysis (G*Power 3.1.9.2, Heinrich-Heine-Universität, Düsseldorf, Germany) using the standard deviation and mean from a previous similar study with a bioabsorbable interference screw by Aga et al. [21] was conducted to determine the sample size. A significance level (α) of .05, a power of 0.8, a standard deviation and an anticipated effect size for ultimate load of 96 N and 120 N, respectively, were used. According to this, a total sample size of 20 was required, so *n* = 10 per group was used, a group size also used by many authors [21–23]. The behaviour of the two screws was compared using a Mann–Whitney U test because normality of data cannot be assumed. When comparing stiffness values at different cycles, Kruskal-Wallis tests were conducted for the same reason. *p*-values ≤ .05 were regarded as significant. The relationship between the insertion torque and yield load was studied by linear regression to obtain the coefficient of determination (*R*^*2*^).

## Results

Insertion torque in tests performed with the 2.5 mm pitch screw group (*n* = 20) was 1.89 ± 0.46 N m, and in the 5 mm pitch screw group (n = 20) was 1.57 ± 0.44 N m, showing no significant difference (*p* = .415).

### 1000 Cycle, 50–250 N tests

One specimen of the 2.5 mm pitch group failed at 11 cycles and one specimen of the 5 mm pitch group failed at 214 cycles. In both cases the failure mode was the pull-out of one strand of the tendon, while the screw remained in its original position. The remaining nine specimens in each of the two screw groups successfully completed the cyclic phase of the test. In the final tensile test the principal failure mode was the pull-out of one or two branches of the tendon; however, in two cases for both screws, the tendon ruptured. The tendon ruptures were associated with higher ultimate loads (887 N and 943 N with the 2.5 mm pitch screw, and 840 N and 863 N with the 5 mm pitch screw). In all cases, no noticeable displacement of the screw was observed.

The results obtained from the nine valid tests are shown in Table 1. As the *p*-values show, no significant differences were found between the two screw groups. Stiffness at the 100th cycle, 1000th cycle, and pull-out showed no significant difference, both for the 2.5 mm pitch (*p* = .565) and for the 5 mm pitch (*p* = .476) screw. The coefficient of determination between insertion torque and yield load (both screws together, *n* = 18) was *R*^*2*^ = .013, indicating that the two variables are not related.

**Table 1:** Results of the 1000 cycles tests for both screws

	2.5 mm pitch	5 mm pitch	*p*
Number of valid samples	9	9	
Maximum insertion torque (N m)	1.74 ± 0.47	1.86 ± 0.34	
Stiffness at 100th cycle (N/mm)	256.4 ± 50.7	259.4 ± 69.4	.757
Stiffness at 1000th cycle (N/mm)	280.3 ± 56.4	275.2 ± 65.0	.965
Displacement at 100th cycle (mm)	1.35 ± 1.15	1.49 ± 2.21	.508
Displacement at 1000th cycle (mm)	6.25 ± 5.66	5.13 ± 4.56	.757
Yield load (N)	482.2 ± 120.2	495.9 ± 131.3	.508
Pull-out stiffness (N/mm)	277.8 ± 71.7	244.5 ± 64.6	.200
Ultimate failure load (N)	656.6 ± 178.7	648.9 ± 169.7	.965

Data are presented as mean ± SD

### 5000 Cycle, 100–300 N tests

One specimen with the 2.5 mm pitch screw failed at 3205 cycles, while four of the specimens with the 5 mm pitch screw failed before the 5000th cycle (failure at 39, 326, 586, and 3344 cycles). The failure mode of these specimens was the pull-out of one or two branches of the tendon. The mode of failure in the specimens tested to pull-out was pull-out of one or two branches of the tendon. The results obtained from the valid tests are shown in Table 2. No significant differences (*p* > .05) were found between the two screw groups, and the coefficient of determination between insertion torque and yield load (*R*^*2*^ = .006, both screws together, *n* = 15) indicated no relationship between the two variables. Stiffness at the 500th cycle, 5000th cycle, and pull-out showed no significant difference, both for the 2.5 mm pitch (*p* = .852) and for the 5 mm pitch (*p* = .459) screw.

**Table 2:** Results of the 5000 cycles tests for both screws

	2.5 mm pitch	5 mm pitch	*p*
Number of valid samples	9	6	
Maximum insertion torque (N m)	2.01 ± 0.45	1.40 ± 0.33	
Stiffness at 500th cycle (N/mm)	270.1 ± 55.7	261.0 ± 69.6	.906
Stiffness at 5000th cycle (N/mm)	281.3 ± 66.4	286.1 ± 79.4	.814
Displacement at 500th cycle (mm)	1.56 ± 0.78	1.84 ± 1.20	.814
Displacement at 5000th cycle (mm)	11.95 ± 14.11	6.97 ± 5.74	.724
Yield load (N)	476.4 ± 65.3	494.3 ± 39.2	.391
Pull-out stiffness (N/mm)	259.9 ± 53.4	236.5 ± 48.6	.886
Ultimate failure load (N)	586.8 ± 44.9	619.1 ± 127.6	.568

Data are presented as mean ± SD

Comparing the 1000 cycle tests with the 5000 cycle tests, no significant difference was found in the yield load for both screws (*p* = .791 and *p* = .556 and for the 2.5 mm pitch and the 5 mm pitch screw, respectively).

## Discussion

The main finding of this study was that the 2.5 mm pitch and the 5 mm pitch screws have the same biomechanical performance under a simulated rehabilitation protocol. Therefore, the thread geometry seems to have no influence on the initial biomechanical properties of an ACL reconstruction with a bioabsorbable interference screw. his conclusion is similar to that obtained with magnesium-based screws [8] and with different buttresses screws [9].

A load protocol of 50–250 N over 1000 cycles represents an aggressive, but typical, rehabilitation protocol [19]. During our tests, 10% of each type of screw failed, which is a similar failure ratio reported by other researchers that used interference screws and similar load protocols [15, 18]. In contrast, a load protocol of 100–300 N over 5000 cycles represents a return to normal daily activities. In these tests, 10% of the 2.5 mm pitch screws and 40% of the 5 mm pitch screws failed, although among those that successfully completed the cyclic testing, there was no significant difference between the two screws. However, the higher failure rate in the 5 mm pitch group leads us to suggest that screws with a very high pitch should not be used if a lack of a rehabilitation program is expected.

Cyclic testing was used to study stiffness and displacement. Stiffness was obtained because the goal of an ACL reconstruction is to restore normal knee kinematics, and matching the intact ACL stiffness is more important than achieving high ultimate failure load [24, 25]. Previous reported stiffness of the intact ACL in young specimens was 242 ± 28 N/mm [26] and 306 ± 80 N/mm [27]. In our tests, stiffness values ranging from 256.4 ± 50.7 N/mm to 286.1 ± 79.4 N/mm were achieved, so both screws are suitable for ACL reconstructions. In addition, the stiffness remained stable during cyclic loading in all tests with both types of screws. Comparing the two screws, no significant differences were observed between the 2.5 mm pitch and 5 mm pitch screw for stiffness values measured at 100 cycles, 1000 cycles, 500 cycles, and 5000 cycles.

Permanent, or residual, displacement of the graft was measured, because it indicates whether there is any increase in laxity of the fixation system as the number of load cycles increases. No significant difference between the displacements of both types of screws was observed. However, large increases in displacement were observed between cycle 100 and 1000, and between cycle 500 and 5000 for both types of screws. Using a similar load protocol (5000 cycles between 50 and 250 N) and a different bioabsorbable interference screw, a previous study reported residual displacements of 9.7 ± 4.9 mm and 10.5 ± 6.1 mm, for screws with diameters of 10 mm and 11 mm, respectively [15]. Smaller displacement at the 1000th cycle in an ACL reconstruction using the 2.5 mm pitch Milagro screw (2.42 ± 1.36 mm) was reported, but a lower load range (20-150 N) was used [28].

Pull-out tests measure the remaining bearing capacity of the fixation system, which is related to the ability of the reconstruction to withstand a traumatic event [29]. Stiffness, yield load, and ultimate failure load were obtained in the pull-out phase of each test. Pull-out stiffness showed no significant difference from the stiffness at the initial (100th or 500th) and the final (1000th or 5000th) cycle, for both types of test and screw. This is expected, since the pull-out test can be considered the last cycle of the test.

Yield load was obtained from the graph because the authors believe that load best represents the fixation system failure load, since elongation increases very rapidly after this load, and may represent clinical failure. This assumption is consistent with a number of other published studies [15, 30–32]. No significant difference was observed in the yield load between the 1000 cycle and 5000 cycle tests, for both types of screw. These results suggest that the ability to withstand load, among those specimens that survived the cyclic phase of the test, is not affected by the number of cycles. The authors believe that the ultimate failure load should not be used to assess a fixation system; however, the ultimate failure load for comparison with other studies has been reported. The authors do not believe that the ultimate failure load is an appropriate measure of failure because it can only be reached at such a high slippage level that a clinical ACL reconstruction would be considered as having already failed.

Insertion torque necessary to place the interference screw is statistically the same with both screws, so the surgeons “feel” the same. However, the use of insertion torque as the fixation strength predictor remains a contentious issue, because some studies have shown that the insertion torque affects the load capacity of a fixation system, [11, 12] while others indicate that there is no relationship between the load and the insertion torque [13, 14]. The relationship between the maximum insertion torque and the yield load was studied and no significant correlation was found. The authors believe that the insertion torque should not be used as an indicator of the quality of the fixation, at least if screw divergence has not been discarded by X-ray images.

The reason why the pitch does not affect the results remains unclear. Theoretically, the higher the pitch, the greater the insertion torque [10] and the lower the pitch, the greater the pull-out strength [33]. However, the results obtained in this study did not show any of those effects, probably because the final bone-screw-graft interface is quite heterogeneous and, therefore, the screw pitch does not play an essential role.

There are some limitations to this study. First, porcine tibiae and bovine digital extensor tendons were used instead of cadaveric specimens. The bovine tendon was used because it has similar biomechanical properties as human double-looped semitendinosus and gracilis grafts [34]. A tibia instead of a femur was used because the tibial fixation site has been reported as the weakest point in ACL reconstructions [21, 35]. The porcine tibia is a widely used model in ACL biomechanical tests because of its availability and because its mechanical properties have greater uniformity than those of the human bones normally available, [7, 21, 36] but concerns exist about its use. It has been suggested that the mechanical properties of a fixation method may not be the same in human tissue as in animal tissue [31]. Another concern is that graft slippage is underestimated and the failure load of the soft tissue graft is overestimated when porcine tibia is utilized compared with young human cadaver tibia [30]. However, like previous studies, [14, 15] the authors believe that since this is a comparative study, the differences between the two screws would also exist in human tissue and the conclusions are therefore valid.

Use of metallic screw was not considered, since this work aimed to compare fixation strength of ACL reconstructions with screws of different pitch and clinical outcomes with titanium and bioabsorbable screws are comparable [2–5].

A second limitation is that this was an in vitro study, so real clinical conditions such as biological osseointegration between bone and graft were not replicated. However, in this study, the main interest was the initial mechanical properties of the ACL reconstruction fixation system and an in vitro study is the gold standard for estimating these properties. Further in vivo studies would be needed to investigate whether there is a difference between the biological behavior of the two types of screws. Another limitation was that pull-out was in the tibial tunnel direction, representing the worst-case scenario, so nothing is known about the possible stress shielding that occurs at the edge of the hole and the exact behavior of the screws during actual flexion-extension knee movement. Again, the authors believe that the conclusions of this study are still valid because the testing conditions for the two screws were the same.

## Conclusions

Thread shape of bioabsorbable interference screws does not seem to affect fixation strength. Despite their significant geometric differences, both interference screws had similar and acceptable biomechanical behavior, so both are suitable to be used in an ACL reconstruction. Therefore, the use of a higher pitch interference screw allows a faster insertion and do not compromise the fixation strength. Insertion torque alone should not be used to estimate the fixation strength because no correlation was found between the insertion torque and the yield load.

## Abbreviations

ACL: Anterior cruciate ligament
Hz: Hertz
mm: Millimeters
MRI: Magnetic resonance imaging
N: Newtons
N/mm: Newtons/millimeter
Nm: Newtons meter
R^*2*^: Coefficient of determination
s: Seconds
SD: Standard deviation
